# The role of environmental factors in sleep disturbances in bipolar disorder: Preliminary analysis from the BEGIN longitudinal study

**DOI:** 10.1192/j.eurpsy.2025.1089

**Published:** 2025-08-26

**Authors:** V. Ruiz, M. Y. Rivas, G. Fico, M. Bort, M. C. Sommerhoff, T. Fernandez, V. Oliva, M. De Prisco, L. Montejo, N. Usachev, N. Lopez Prieto, S. Garcia-Diaz, M. D. P. Marton, L. Maitre, E. Vieta, A. Murru

**Affiliations:** 1 Department of Pyschiatry, Hospital Clinic of Barcelona; 2 Bipolar and Depressive Disorders Unit, Hospital Clinic de Barcelona, Barcelona, Spain; 3 Pyschiatry, Bipolar and Depressive Disorders Unit, Hospital Clinic de Barcelona; 4 Barcelona Institute for Global Health (ISGlobal); 5 Department of Pyschiatry, Bipolar and Depressive Disorders Unit, Hospital Clinic de Barcelona, Barcelona, Spain

## Abstract

**Introduction:**

Sleep disturbances are common among individuals with bipolar disorder (BD) and may be present even during euthymic phases and significantly impact illness course and quality of life. This preliminary analysis is part of BEGIN (The Bipolar Exposome-Gene Interaction Naturalistic study.

**Objectives:**

Here, we analyze the relationship between subjective sleep disturbances and various lifestyle and environmental factors in BD patients.

**Methods:**

Eighty-seven patients diagnosed with BD (Mean age = 50.7 years, SD = 13.6; 44.7% female; Mean BMI = 26.9, SD = 4.61) all in a three-month euthymic phase, were recruited. At baseline, participants completed lifestyle questionnaires covering diet, light exposure, and time spent indoors, alongside assessments of clinical history and circadian rhythms using the BRIAN scale. Sleep quality was assessed through the Pittsburgh Sleep Quality Index (PSQI, with a cutoff >5 for sleep problems) and the Epworth Sleepiness Scale (ESS, with a cutoff >10 indicating excessive daytime sleepiness). Sociodemographic, clinical, and environmental factors were compared between BD patients with and without sleep disturbances.

**Results:**

Based on ESS scores, patients with excessive daytime sleepiness had significantly higher overall circadian rhythm disruptions, as indicated by the BRIAN total score (p = 0.049). A negative correlation emerged between excessive daytime sleepiness and age at first hospitalization (r = -0.36, p = 0.003). Disrupted eating patterns, reflected in the BRIAN eating subscale, also correlated with excessive daytime sleepiness (r = 0.27, p = 0.027). Based on PSQI scores tobacco smoking was positively associated with poor sleep quality (r = 0.42, p = 0.013), while more time spent with artificial light from electronic devices (r = -0.29, p = 0.019), and less time spent indoors (r = 0.39, p = 0.001) correlated with worse sleep quality. Patients experiencing poor sleep also showed less consistency in social routines (BRIAN social score, p = 0.028).

**Conclusions:**

These preliminary findings suggest that sleep disturbances in BD patients may be intricately linked to lifestyle and environmental factors, such as circadian rhythm disruptions, smoking, and exposure to artificial light. These results highlight the importance of considering environmental and lifestyle modifications to support sleep quality in BD. Future longitudinal analyses will be essential to clarify causal pathways and develop targeted interventions that address circadian and lifestyle factors in managing BD.

**Disclosure of Interest:**

None Declared

